# Changing Emergence of *Shigella* Sero-Groups in Bangladesh: Observation from Four Different Diarrheal Disease Hospitals

**DOI:** 10.1371/journal.pone.0062029

**Published:** 2013-04-29

**Authors:** Sumon Kumar Das, Shahnawaz Ahmed, Farzana Ferdous, Fahmida Dil Farzana, Mohammod Jobayer Chisti, Daniel T. Leung, Mohammad Abdul Malek, Kaisar Ali Talukder, Pradip Kumar Bardhan, Mohammed Abdus Salam, Abu Syed Golam Faruque, Rubhana Raqib

**Affiliations:** 1 Centre for Nutrition and Food Security (CNFS), International Centre for Diarrhoeal Disease Research, Bangladesh (icddr,b), Dhaka, Bangladesh; 2 Centre for Vaccine Science (CVS), International Centre for Diarrhoeal Disease Research, Bangladesh (icddr,b), Dhaka, Bangladesh; 3 Centre for Food and Water Disease (CFWD), International Centre for Diarrhoeal Disease Research, Bangladesh (icddr,b), Dhaka, Bangladesh; 4 Dhaka Hospital, International Centre for Diarrhoeal Disease Research, Bangladesh (icddr,b), Dhaka, Bangladesh; 5 Research Administration Services (RAS), International Centre for Diarrhoeal Disease Research, Bangladesh (icddr,b), Dhaka, Bangladesh; Beijing Institute of Microbiology and Epidemiology, China

## Abstract

**Background:**

Shigellosis continues to be a public health challenge for developing countries, including Bangladesh. The aim of the study is to demonstrate recent changes in *Shigella* sero-groups and their geographical diversity.

**Methods:**

Data were extracted from data archive of four diarrheal disease surveillance systems. A 2% sub sample from urban Dhaka Hospital (2008–2011; n = 10,650), and 10% from urban Mirpur Treatment Centre (2009–2011; n = 3,585), were enrolled systematically; whereas, all patients coming from the Health and Demographic Surveillance System area in rural Matlab (2008–2011; n = 6,399) and rural Mirzapur (2010–2011; n = 2,812) were included irrespective of age, sex, and disease severity. A fresh stool specimen was collected for identification of *Shigella* spp. Of them, 315 (3%) were positive for *Shigella* in Dhaka, 490 (8%) from Matlab, 109 (3%) from Mirpur and 369 (13%) from Mirzapur and considered as analyzable sample size.

**Results:**

Among all *Shigella* isolates regardless of age, significant decreases in percentage of *S. flexneri* over time was observed in Mirpur (55→29%; p value of χ^2^-for trend = 0.019) and Mirzapur (59→47%; p = 0.025). A non-significant decrease was also seen in Dhaka (58→48%), while in Matlab there was a non-significant increase (73→81%). Similar patterns were observed among under-5 children at all sites. Emergence of *S. sonnei* was found in Dhaka (8→25%; p<0.001) and Mirpur (10→33%; p = 0.015), whereas it decreased in Mirzapur (32→23%; p = 0.056). The emergence of *S. boydii* was seen in all ages in Mirzapur [(3→28%; p<0.001); (3→27%; p<0.001)]. On the other hand, we saw non-significant percent reductions in *S. boydii* in Dhaka [overall (25→16%); under-5 (16→9%)]. Decreasing rates *of Shigella dysenteriae* were observed in Matlab, Mirpur and Mirzapur; whereas, in Dhaka it remained unchanged.

**Conclusion and Significance:**

Emergence of *S. sonnei* and *S. boydii* as important infectious diarrhea etiologies and variations in geographical diversity underscore the need for monitoring, with possible implications for vaccine development.

## Introduction

Shigellosis, a food-borne illness caused by the genus *Shigella*, is usually transmitted through person-to-person contact [1]. It is one of the most common causes of childhood dysentery and an important infectious cause of morbidity and mortality in children under 5 years of age. Annually, approximately 90 million cases occur with at least 100,000 deaths, mostly (60%) among children under five years of age in developing countries [2]. *S. flexneri, S. sonnei, S. boydii* and *S. dysenteriae*, the four species or sero-groups, based on biochemical properties and group-specific O antigens in the outer membrane of the cell wall, are responsible for the disease [3]. The disease is characterized by a short period of watery diarrhea with intestinal cramps and general malaise, soon followed by emission of bloody, mucoid, often mucopurulent stools [2]. A population-based prospective multi-country study in Asia aiming to get a better understanding of the current disease burden, clinical manifestations, and microbiology of shigellosis revealed that *Shigella* appears to be more universal in Asian impoverished populations than previously estimated and antibiotic-resistant strains of different species and serotypes have emerged [4].

Like other *Shigella* prevalent countries, Bangladesh is considered an endemic zone of shigellosis. A recent study of isolation patterns of different sero-groups of *Shigella* in a large diarrheal disease hospital in Dhaka showed a decreased proportion of shigellosis from 8–12% in the 1980s to 3% in 2008 [5]. This study also documented the interquartile range of isolation rate of the sero-groups over the period as *S. flexneri* 46–63%, *S. dysenteriae* 0–25%, *S. boydii* 10–21% and *Shigella sonnei* 4–9% [5]. However, there is a paucity of data on the rate of change of specific sero-groups. Furthermore, data is lacking regarding the isolation of *Shigella* in rural versus urban settings. Knowledge on the changing patterns of isolation of *Shigella* sero-groups and their geographical diversity have potential public health implications [6]. Thus, our aim was to demonstrate the changing pattern of *Shigella* sero-groups and their geographical diversity in four different diarrheal disease treatment facilities in Bangladesh.

## Materials and Methods

### Study sites

#### Dhaka Hospital – Dhaka

**Figure 1:**
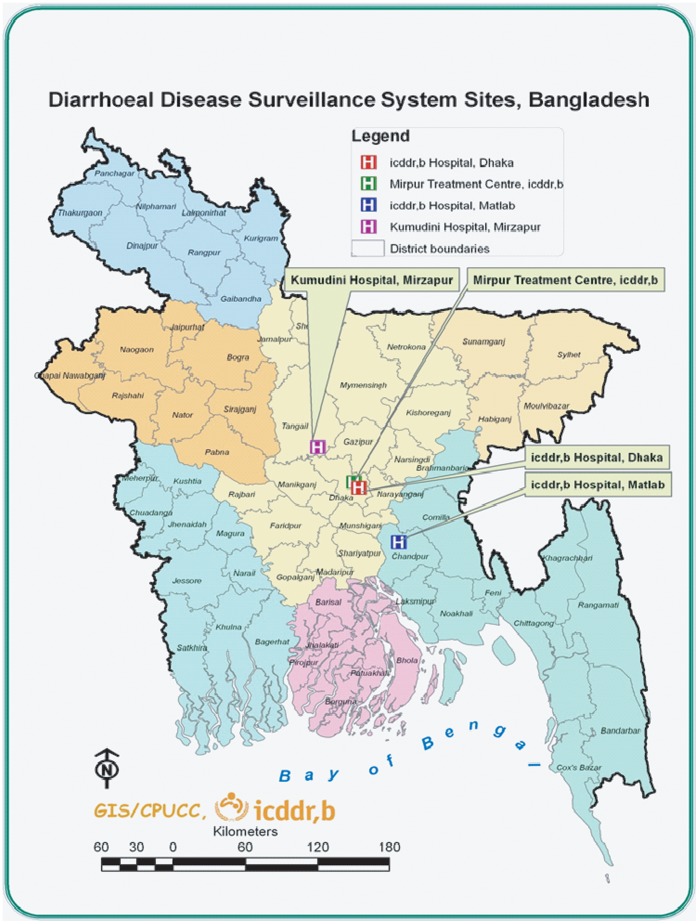
Study sites.

The Dhaka Hospital of International Centre for Diarrheal Disease Research, Bangladesh (icddr,b) offers free treatment to at least 140,000 people a year. A diarrheal disease surveillance system (DDSS) was established in 1979. The urban DDSS currently collects information on the clinical, epidemiological and demographic characteristics, feeding practices (particularly of infants and young children), and use of drug and fluid therapy at home of every 50^th^ patient, irrespective of age, sex, disease severity or socioeconomic status by administering structured questionnaire. The generated data provides valuable information to hospital clinicians in their decision-making processes and enables the detection of emerging pathogens and early identification of outbreaks and their locations as well as antimicrobial susceptibility pattern (Figure 1).

#### Matlab Hospital-Matlab

ICDDR,B maintains one of the richest, most comprehensive and longest running, longitudinal data resources in the developing world, producing regular accurate demographic and health data for rural Matlab, Bangladesh. With over 40 years of continuous demographic information on a population of over 200,000 people, Matlab is our major rural field site, and a major public health resource for the world. The site is a model for public health strategies around the world. The Health and Demographic Surveillance System at Matlab covers a population of about 225,000, providing data necessary to plan, conduct, and evaluate various types of public-health intervention research. Since 1963, icddr,b has been maintaining a treatment facility in rural Matlab (located about 55 km from Dhaka) for treating diarrhea patients. It provides free treatment to 12,000–15,000 diarrhea patients annually reporting from Health and Demographic Surveillance System area and other adjoining sub-districts (Figure 1).

#### Mirpur treatment Centre-Mirpur

The Mirpur area is in the north-west of the Dhaka, the capital city. This 60-bed urban hospital at Mirpur began it’s operations in April, 2009. More than 12,000 patients annually receive care in this facility. Every 10^th^ patient presenting to the facility is included into the surveillance system (Figure 1).

#### Kumudini Hospital – Mirzapur

Mirzapur sub-district covers 374 km^2^ in Tangail district (Figure 1), and is located about 60 km north-west of Dhaka. It has a total of 58,300 households and a population of 238,463, according to a 2007 census. Almost all households have access to a tube well as a source of drinking water; around half have hygienic sanitary toilets, and 60% have electricity. Agriculture is the main occupation for men, while women work mainly in the home. Kumudini Hospital is located in the middle of the Demographic Surveillance System (DSS) area, is a philanthropic institution established in 1938 in Mirzapur (under Tangail District) providing health services to the surrounding poor rural population. Since 1982, a separate diarrhea treatment unit was started that serves nearly 1,500 diarrhea patients each year for treatment (Figure 1).

### Sample Framing

A 2% sub sample from Dhaka Hospital and 10% from Mirpur Treatment Centre were enrolled systematically; whereas, all patients coming from the Health and Demographic Surveillance System area in Matlab and Mirzapur were included irrespective of age, sex, and disease severity. We extracted data from the data archive of the different diarrheal disease surveillance system as follows: Dhaka Hospital (2008–2011; n = 10,650), Matlab (2008–2011; n = 6,399), Mirpur (2009–2011; n = 3,585), and Mirzapur (2010–2011; n = 2,812) (Table 1).

**Table 1:** Distribution of overall isolation rate of *Shigella* in four different diarrheal treatment centres.

	Overall				<5 years			
Year	Dhaka	Matlab	Mirpur	Mirzapur	Dhaka	Matlab	Mirpur	Mirzapur
2008	80/2444 (3)	184/1996 (9)^a^	NA	NA	46/1198 (4)	108/1340 (8)^a^	NA	NA
2009	82/2825 (3)	112/1532 (7)^a^	29/582 (5)	NA	42/1236 (3)	73/874 (8)^a^	12/232 (5)	NA
2010	86/2853 (3)	95/1547 (6)^a^	38/1485 (3)^c^	196/1287 (15)^abde^	43/1345 (3)	47/742 (6)^a^	19/586 (3)^cd^	175/1039 (17)^abde^
2011	67/2528 (3)	99/1324 (8)^a^	42/1518 (3)^dc^	173/1525 (11)^abde^	35/1296 (3)	46/679 (7)^a^	17/666 (3)^cd^	153/1278 (12)^abde^

Note: Data described as number of isolates/total samples tested (results considered statistical significant at 5% level).

a Comparison between Dhaka and Matlab.

b Comparison between Dhaka and Mirzapur.

c Comparison between Matlab and Mirpur.

d Comparison between Matlab and Mirzapur.

e Comparison between Mirpur and Mirzapur.

### Specimen Collection and Laboratory Procedure

A single fresh whole stool specimen (at least 3 ml/grams) were collected. Maintaining cool temperature (+4 to +8 degree Celsius), all specimens from Dhaka, Mirpur and Mirzapur were submitted to the central laboratory in Dhaka within 6 hours of collection in Carry - Blair media. Stool samples from Matlab were processed in the Matlab Microbiology Laboratory. *Shigella* spp. [7] were isolated using standard methods.

### Data Management and Analysis

Statistical Package for Social Sciences (SPSS) Windows (Version 15.2; Chicago, IL) was used for data entry and subsequent analysis. Descriptive analysis was employed and categorical variables were expressed in proportion. Chi-square for trend was performed to determine the changing trend of *Shigella* sero-groups over the study period provided that the observation period was of three or more years. However, for less than three years (Mirzapur) Chi-square test was equated to determine any statistical difference with a probability value of <0.05.

### Ethical Statement

The Diarrhoeal Disease Surveillance System (DDSS) of icddr,b is a routine ongoing activity of the Dhaka Hospital, Matlab Hospital and Mirpur Treatment Centre which has been approved by the Research Review Committee (RRC) and Ethical Review Committee (ERC) of icddr,b. At the time of enrollment, verbal consent was taken from the caregivers or guardians on behalf of the patients. The information was stored in the hospital database and used for conducting researches. The DDSS of icddr,b is a scheduled activity on the hospital patients, and used to be performed after taking verbal consent from the parents or guardians of the patients following the hospital policy. Parents or guardians were assured about the non-disclosure of information collected from them, and were also informed about the use of data for analysis and using the results for improving patient care activities as well as publication without disclosing the name or identity of their children. ERC was satisfied with the voluntary participation, maintenance of the rights of the participants and confidential handling of personal information by the hospital physicians and has approved this consent procedure. However, the surveillance activities in Mirzapur Kumudini Hospital has also been approved by the RRC and ERC of icddr,b. Both informed written consent as well as accent has been taken from the study population. Thus, verbal consent is taken from patients enrolled in Dhaka, Matlab and Mirpur surveillance system and for Mirzapur, written consent from adults or guardians and assent from 11–17 years old children has been taken.

## Results

Overall isolation of *Shigella* was higher in rural Mirzapur compared to other sites. However, a higher rate was also observed in rural Matlab in contrast to the two urban sites (Table 1). Among children under 5 years, such observation remained similar between sites over the period.

Distinct socio-demographic diversity among individuals infected with *Shigella* was observed across the sites. In urban Dhaka and Mirpur, *Shigella* was more often detected among males compared to that of rural Matlab and Mirzapur. Maternal literacy rate was also higher in urban sites. Considering poor socio-economic status, lesser proportion of individuals with shigellosis belonged to Mirpur compared to others sites. Proportion of individuals having access to sanitary toilets and used antimicrobials before hospital visit was low in rural Matlab. On the other hand, urban individuals with shigellosis often presented with watery stool and some or severe dehydration compared to rural sites. However, rural residents had often abdominal pain and fever. Duration of diarrhea, vomiting and nutritional status (underweight, stunting and wasting) were found identical across the sites (Table 2).

**Table 2:** Overall Characteristics of patients with *Shigella* in four diarrhoeal treatment facilities of Bangladesh.

Characteristic	Dhaka; n = 315 (%)	Matlab; n = 490 (%)	p-value	Mirpur; n = 109 (%)	p-value	Mirzapur; n = 369 (%)	p-value
Male	192 (61)	252 (52)	0.009^a^	70 (64)	0.623^a^/0.020^b^	206 (56)	0.216^a^/0.210^b^/0.155^c^
Illiterate mother	51/188 (27)	26/306 (9)	<0.001^a^	12/58 (21)	0.417^a^/0.010^b^	34/337 (9)	0.008^a^/0.577^b^/0.035^c^
Poor socio-economic status (median monthlyfamily income<US$100)	161 (51)	284 (58)	0.066^a^	10 (9)	<0.001^a^/<0.001^b^	160 (44)	0.055^a^/<0.001^b^/<0.001^c^
Use of sanitary toilet	252 (80)	62 (13)	<0.001^a^	67 (62)	<0.001^a^/<0.001^b^	275 (75)	0.122^a^/<0.001^b^/0.562^c^
Use of antimicrobial prior to hospital visit	101 (32)	97 (20)	<0.001^a^	30 (26)	0.444^a^/0.097^b^	116 (32)	0.944^a^/<0.001^b^/0.498^c^
Watery stool (lack of mucus/blood)	275 (87)	225 (46)	<0.001^a^	102 (94)	0.104^a^/<0.001^b^	125 (34)	<0.001^a^/<0.001^b^/0.264^c^
Duration of diarrhoea (<1 day)	124 (39)	222 (45)	0.112^a^	50 (46)	0.281^a^/0.999^b^	121 (33)	0.092^a^/<0.001^b^/0.017^c^
Dehydration (moderate/severe)	194 (62)	95 (19)	<0.001^a^	65 (60)	0.805^a^/<0.001^b^	64 (17)	<0.001^a^/0.511^b^/<0.001^c^
Abdominal pain	174 (55)	354 (72)	<0.001^a^	51 (47)	0.157^a^/<0.001^b^	300 (82)	<0.001^a^/0.002^b^/<0.001^c^
Vomiting	191 (61)	267 (55)	0.099^a^	69 (63)	0.704^a^/0.116^b^	171 (47)	<0.001^a^/0.023^b^/0.002^c^
Fever (≥37.8°C)	31 (10)	121 (25)	<0.001^a^	6 (6)	0.235^a^/<0.001^b^	131 (36)	<0.001^a^/<0.001^b^/<0.001^c^
Underweight	63/166 (38)	101/274 (37)	0.898^a^	17/44 (39)	0.927^a^/0.953^b^	97/328 (30)	0.062^a^/0.070^b^/0.293^c^
Stunting	45/166 (27)	84/274 (31)	0.478^a^	11/44 (25)	0.928^a^/0.559^b^	71/328 (22)	0.101^a^/0.015^b^/0.756^c^
Wasting	38/166 (23)	61/274 (22)	0.971^a^	12/44 (27)	0.683^a^/0.588^b^	64/328 (20)	0.065^a^/0.466^b^/0.317^c^

a Comparison between Dhaka with other three sties (results considered statistical significant at 5% level).

b Comparison between Matlab with Mirpur and Mirzapur sties (results considered statistical significant at 5% level).

c Comparison between Mirpur with Mirzapur stie (results considered statistical significant at 5% level).

**Figure 2:**
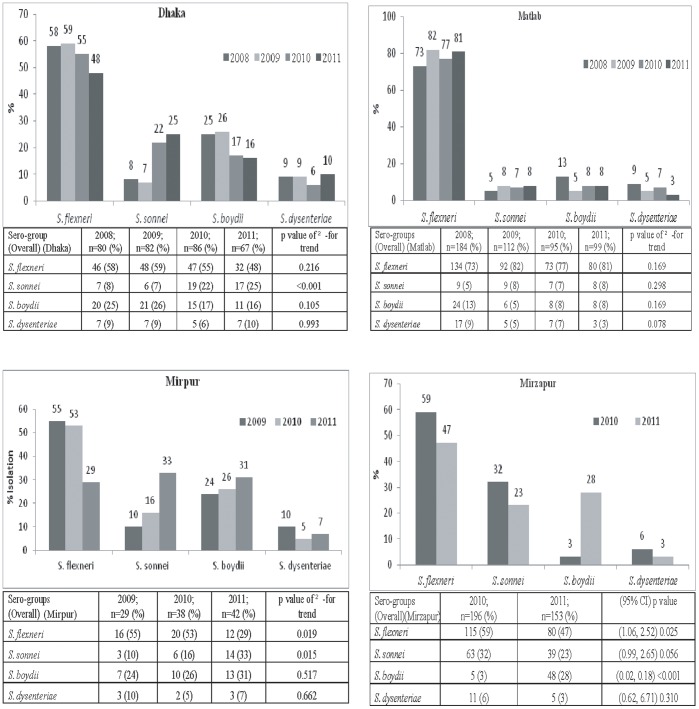
Overall yearly isolation of *Shigella* by sero-goups in different sites.

**Figure 3:**
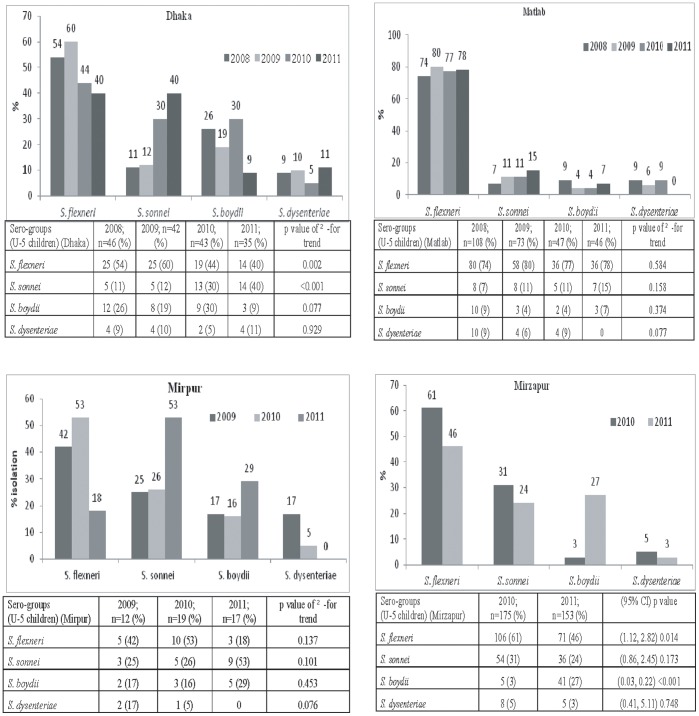
Yearly isolation of *Shigella* by sero-goups among under 5 children in different sites.

In Dhaka, of all *Shigella* isolates, the proportion of *–S. flexneri* in 2008 was 58% which gradually decreased to 48% in 2011 (Figure 2). The proportion of *S. flexneri* among total *Shigella* isolated in <5 years old children also decreased (Figure 3). Similarly, in the Mirpur Treatment Centre, there was a decreasing trend of *S. flexneri* in all age groups as well as among under-5 children in 2009–11 (Figure 2, 3). Decreasing trend in proportion of *S. flexneri* was also observed in Mirzapur during 2010 and 2011 (Figure 2). Among the under-5 years old children, similar change in proportion was found in Dhaka and Mirzapur (Figure 3). On the other hand, in Matlab, the distribution of *S. flexneri* was similar over the 4-year period (Figure 2) which remained the highest isolate among all the *Shigella* spp. Among the under 5 children, the scenario was similar (Figure 3).

In contrast to *S. flexneri*, proportion of *S. sonnei* among all *Shigella* isolates increased steadily - in all age groups in the Dhaka Hospital and Mirpur Treatment Centre (Figure 2). In line with this, among the under-5 children the proportion also increased substantially (Figure 3). In Matlab, the isolation rate of *S. sonnei* remained static in all age groups (Figure 2); although in under-5 children, the proportion increased two folds (Figure 3). In Mirzapur, the prevalence of *S. sonnei* declined somewhat in all age groups as well as in under-5 children (Figure 2, 3).

The proportion of *S. boydii* was higher in 2008 that decreased over years in 2011 in both Dhaka Hospital and in Matlab (Figure 2). A similar trend was noted in the under-5 children (Figure 3). However, the proportion of *S. boydii* among all *Shigella* isolates in Mirpur and Mirzapur (Figures 2, 3) was the opposite with dramatic increase in the isolation rates, especially in the under 5 children. Proportion of *S. dysenteriae* remained the same over the 4-year period in Dhaka, Matlab and Mirzapur (Figure 2, 3). Interestingly, in the Mirpur Treatment Center there was no isolation of *S. dysenteriae* in the year 2011 among children less than 5 years of age (Figure 3).

## Discussion

The observation of increasing rates of infectious diarrhea caused by *S. sonnei* and *S. boydii* and decreasing rates of *S. flexneri* in four different diarrheal disease facilities has important public health implications. *S. sonnei* is one of the primary causes of dysentery in developed countries, and is now replacing *S. flexneri* as the leading cause of the illness in many developing countries or countries in transition [4] [8], [9], [10]. It has been hypothesized that these changes may be due to varying host characteristics such as reduction of overall malnutrition that in turn leads to improved immune response. It may also be due to genetic mutation of the non-virulent microbes becoming pathogenic [11]. Moreover, changing meteorological indicators, such as rising temperatures, decreased rainfall, increased sea level and temperature [10]; irrational use and increased minimum inhibitory concentration (MIC) level to common antimicrobials [12], [13] and disappearance of epidemic strains such as *S. dysenteriae* type 1, resulting in resurgence of non-epidemic strains as hyperendemic strains, may also have contributed [5], [14].

*Shigella* spp. are dynamic and able to survive in a range of ecological niches under diverse environmental conditions. Recent phylogenetic analysis showed that the current *S. sonnei* population descends from a common ancestor that existed about 500 years ago and that diversified into several distinct lineages with unique characteristics which mainly occurred in Europe [8]. Such mutations may also play a role in our context, and deserves further study. Shigellosis due to *S. sonnei* is reported to occur at low rates among people living in developing countries. *Plesiomonas shigelloides* is a gram-negative bacteria often found in surface water that shares antigens with *S. sonnei*, and until recently *P. shigelloides* has been a frequently isolated enteric pathogen from <2 years old Bangladeshi children in the diarrhea hospital [15], [16]. It is well accepted that exposure to *P shigelloides,* through contaminated drinking water, may immunize people to *S. sonnei* in these developing countries [17]. Increased and sustained access to safe drinking water, one of the major components of Millennium Development Goals to control and prevent food and water borne diseases, is a major step that is taken into account by the policy makers of transitional countries. With economic development and accompanying water quality improvements, susceptibility to *S. sonnei* in a population may increase [8], [17]. Bangladesh is a country in transition and the national GDP per capita of Bangladesh increased from US$ 356 to 674 from 2008 to 2010 [18]. This gross economic improvement might have impacted positively on the overall behavioral change in general population with more and more of the population using bottled water in urban areas and having access to piped water in comparison to rural areas. The changing trend in the serotype distribution of *Shigella* spp. may reflect this phenomenon. In the rural community such as Matlab, the distribution of the different species of *Shigella* was different than that in urban areas (Dhaka, Mirpur) suggesting differences in life style, access to different types of water and food, and local environmental conditions.

The heterogeneous distribution of *Shigella* serotypes in urban and rural Bangladesh suggests that multivalent vaccines will be needed to prevent shigellosis in these settings. Currently, there are no licensed vaccines available for the prevention of shigellosis, especially given the wide range of *Shigella* serotypes as well as undifferentiated strains that differ from the known available strains [19]. Therefore, the findings of the study underscore the need for a multivalent *Shigella* vaccine for the prevalent species and serotypes.

All together, further investigations on factors such as host and environmental characteristics, and climate change need to be prioritized to address the phenomenon. Meanwhile, surveillance systems play a critical role in monitoring the changes of the sero-groups and serotypes of *Shigella*. Furthermore, such systems may identify factors to predict outbreaks of disease as well as changes in antimicrobial susceptibility patterns.

### Limitations

Hospital based data may not adequately represent the sick population at large in the community. However, our strengths were that we followed an unbiased systematic sampling irrespective of age, sex, nutrition status, disease severity or socioeconomic context, observed isolation patterns in different geographical locations, and used a large and longitudinal data set for this analysis. Moreover, despite variations over the study period in different sites, some differences however were not statistically significant possibly due to lack of power with small sample size.

## References

[1] Clemens J, Kotloff K, Kay B (1999) Generic protocol to estimate the burden of Shigella diarrhoea and dysenteric mortalit.World Health Organization: Department of Vaccines and Biologicals. Geneva: World Health Organization.

[2] WHO (2009) Initiative for Vaccine Research. Diarrhoeal Diseases (Updated February 2009): Shigellosis. Geneva: World Health Organization.

[3] Hale TL, Keusch GT (1996) Shigella. Medical Microbiology. Galveston (TX): University of Texas Medical Branch at Galveston: Baron S, editor. 21413292

[4] von Seidlein L, Kim DR, Ali M, Lee H, Wang X, et al (2006) A multicentre study of Shigella diarrhoea in six Asian countries: disease burden, clinical manifestations, and microbiology. PLoS Med 3: e353. 16968124 10.1371/journal.pmed.0030353 PMC1564174

[5] Khatun F, Faruque AS, Koeck JL, Olliaro P, Millet P, et al (2011) Changing species distribution and antimicrobial susceptibility pattern of Shigella over a 29-year period (1980–2008). Epidemiol Infect 139: 446–452. 20478088 10.1017/S0950268810001093

[6] Vinh H, Nhu NT, Nga TV, Duy PT, Campbell JI, et al (2009) A changing picture of shigellosis in southern Vietnam: shifting species dominance, antimicrobial susceptibility and clinical presentation. BMC Infect Dis 9: 204. 20003464 10.1186/1471-2334-9-204 PMC2803792

[7] World Health Organization (1987) Programme for control of diarrheal disease. In Manual for laboratory investigation of acute enteric infections. Geneva, Switzerland: World Health Organization. 9–20 p.

[8] Holt KE, Baker S, Weill FX, Holmes EC, Kitchen A, et al (2012) Shigella sonnei genome sequencing and phylogenetic analysis indicate recent global dissemination from Europe. Nat Genet 44: 1056–1059. 22863732 10.1038/ng.2369 PMC3442231

[9] Chompook P, Samosornsuk S, von Seidlein L, Jitsanguansuk S, Sirima N, et al (2005) Estimating the burden of shigellosis in Thailand: 36-month population-based surveillance study. Bull World Health Organ 83: 739–746. 16283050 PMC2626425

[10] Kelly-Hope LA, Alonso WJ, Thiem VD, Anh DD, Canh do G, et al (2007) Geographical distribution and risk factors associated with enteric diseases in Vietnam. Am J Trop Med Hyg 76: 706–712. 17426175

[11] Greenhill C (2012) Infectious disease: Genome sequencing reveals how Shigella sonnei spread around the world. Nat Rev Gastroenterol Hepatol 9: 487. 22907165 10.1038/nrgastro.2012.160

[12] Christopher PR, David KV, John SM, Sankarapandian V (2010) Antibiotic therapy for Shigella dysentery. Cochrane Database Syst Rev: CD006784. 10.1002/14651858.CD006784.pub3 20091606

[13] Vinh H, Anh VT, Anh ND, Campbell JI, Hoang NV, et al (2011) A multi-center randomized trial to assess the efficacy of gatifloxacin versus ciprofloxacin for the treatment of shigellosis in Vietnamese children. PLoS Negl Trop Dis 5: e1264. 21829747 10.1371/journal.pntd.0001264 PMC3149021

[14] Feil EJ (2012) The emergence and spread of dysentery. Nat Genet 44: 964–965. 22932498 10.1038/ng.2389

[15] Khan AM, Faruque AS, Hossain MS, Sattar S, Fuchs GJ, et al (2004) Plesiomonas shigelloides-associated diarrhoea in Bangladeshi children: a hospital-based surveillance study. J Trop Pediatr 50: 354–356. 15537721 10.1093/tropej/50.6.354

[16] Khan AM, Hossain MS, Khan AI, Chisti MJ, Chowdhury F, et al (2009) Bacterial enteropathogens of neonates admitted to an urban diarrhoeal hospital in Bangladesh. J Trop Pediatr 55: 122–124. 18840632 10.1093/tropej/fmn090

[17] Sack DA, Hoque AT, Huq A, Etheridge M (1994) Is protection against shigellosis induced by natural infection with Plesiomonas shigelloides? Lancet 343: 1413–1415. 7910890 10.1016/s0140-6736(94)92531-3

[18] Index Mundi website. Available: http://www.indexmundi.com/facts/indicators/NY.GDP.PCAP.PP.CD. Accessed 2012 Dec 5.

[19] Kaminski RW, Oaks EV (2009) Inactivated and subunit vaccines to prevent shigellosis. Expert Rev Vaccines 8: 1693–1704. 19943764 10.1586/erv.09.127

